# Differentially Expressed miRNAs in Tumor, Adjacent, and Normal Tissues of Lung Adenocarcinoma

**DOI:** 10.1155/2016/1428271

**Published:** 2016-05-10

**Authors:** Fei Tian, Rui Li, Zhenzhu Chen, Yanting Shen, Jiafeng Lu, Xueying Xie, Qinyu Ge

**Affiliations:** ^1^Research Center for Learning Science, Southeast University, Nanjing 210096, China; ^2^State Key Lab of Bioelectronics, Southeast University, Nanjing 210096, China

## Abstract

Lung cancer is the leading cause of cancer deaths. Non-small-cell lung cancer (NSCLC) is the major type of lung cancer. The aim of this study was to characterize the expression profiles of miRNAs in adenocarcinoma (AC), one major subtype of NSCLC. In this study, the miRNAs were detected in normal, adjacent, and tumor tissues by next-generation sequencing. Then the expression levels of differential miRNAs were quantified by quantitative reverse transcription-polymerase chain reaction (qRT-PCR). In the results, 259, 401, and 389 miRNAs were detected in tumor, adjacent, and normal tissues of pooled AC samples, respectively. In addition, for the first time we have found that miR-21-5p and miR-196a-5p were gradually upregulated from normal to adjacent to tumor tissues; miR-218-5p was gradually downregulated with 2-fold or greater change in AC tissues. These 3 miRNAs were validated by qRT-PCR. Lastly, we predicted target genes of these 3 miRNAs and enriched the potential functions and regulatory pathways. The aberrant miR-21-5p, miR-196a-5p, and miR-218-5p may become biomarkers for diagnosis and prognosis of lung adenocarcinoma. This research may be useful for lung adenocarcinoma diagnosis and the study of pathology in lung cancer.

## 1. Introduction

Lung cancer is the leading cause of cancer-related deaths in the world [1]. Non-small-cell lung cancer (NSCLC) is the main type of lung cancer, which accounts for 80–85%. NSCLC mainly consists of squamous cell carcinoma (SCC) and adenocarcinoma (AC) [2]. Patients with NSCLC were frequently diagnosed at advanced stages, resulting in an overall 5-year survival rate of about 14% [3]. In order to improve the outcome of these patients, the early diagnosis of NSCLC becomes necessary. In recent years, studies showed that molecular biomarkers were discovered in various cancers and could be applied in the diagnosis and prognosis of NSCLC [4–6].

MicroRNAs (miRNAs) are endogenous noncoding small RNAs of about 18–25 nucleotides [7]. Mature miRNAs are highly conserved RNA molecules that can regulate the expression of genes by hybridizing to complementary sequences in the 3′-untranslated (UTR) region of target mRNAs [8]. Studies have shown that miRNAs participate in a lot of biological processes, including cell proliferation, cell differentiation, and cell apoptosis [9]. In addition, a large number of studies have shown that miRNAs may play roles in the tumorigenesis and development of lung cancer as tumor oncogenes or suppressor genes [10–14]. However, few researchers studied the expression profiles of miRNAs in paired tumor, adjacent, and normal tissues of lung cancer.

Three principal methods can be adopted to detect the expression levels of miRNAs: quantitative reverse transcription-polymerase chain reaction (qRT-PCR), microarray, and next-generation sequencing (NGS). Quantitative RT-PCR and microarray can only detect relative expression levels of known miRNAs, and the number of analyzed miRNAs is limited. Compared to qRT-PCR and microarray, NGS has an advantage in global miRNA analysis and it provides the possibility of detecting novel miRNAs. What is more, the analysis of miRNAs becomes faster and cheaper based on NGS [15].

In this study, the miRNAs were detected in tumor, adjacent, and normal tissues from AC pooled samples by NGS. The differential miR-21-5p, miR-196a-5p, and miR-218-5p were validated by qRT-PCR. The purpose of this study was to systematically characterize the expression of miRNAs in AC tumor, adjacent, and normal tissues. miR-21-5p, miR-196a-5p, and miR-218-5p can be biomarkers for diagnosis and prognosis of AC. The gradually changed miRNAs may be useful for AC diagnosis and the study of pathology in lung cancer.

## 2. Material and Method

### 2.1. Sample Preparation and RNA Isolation

We collected 18 surgically resected samples including paired tumor, adjacent, and normal lung tissues from 18 AC patients. Tissue samples were all obtained from Jiangsu Province Hospital with informed consent. Tissues were stored at −80°C and then ground for subsequent RNA isolation.

Total RNA was isolated from 50 mg tissue of each sample by mirVana*™* miRNA Isolation Kit (Ambion, USA). The quantity and quality of obtained RNA were measured by Qubit® 2.0 Fluorometer (Life Technologies, USA). The total RNA isolated from 9 AC tumor tissue samples was pooled with equimolar amounts, respectively, and the same operation was applied to 9 AC adjacent and 9 normal tissues. The miRNAs of the 3 pooled samples were analyzed by next-generation sequencing. Another 9 paired AC samples were used for following qRT-PCR validation.

### 2.2. Small RNA Library Preparation for Ion Torrent Sequencing

The small RNA libraries for Ion Torrent sequencing were prepared according to the manufacturer's protocols of Ion Total RNA-Seq Kit v2 (Life Technologies, USA) and Ion Xpress*™* RNA-Seq Barcode 1-16 Kit (Life Technologies, USA). The libraries were sequenced by Ion Torrent Proton*™* sequencing platform after quality control at Nanjing Percare Biotechnology Co., Ltd.

### 2.3. Bioinformation Analysis

The raw expression values (read counts) of Ion Torrent were filtered by quality control. The effective reads were analyzed through four steps. Firstly, the basic results were obtained, including the length distribution, mapping to reference genome, mapping to rfam, and the repeat distribution. Secondly, miRNAs (including novel miRNAs) were predicted. Thirdly, miRNA target genes were predicted and annotated. Lastly, other ncRNAs were annotated, including tRNA, rRNA, snoRNA, snRNA, and piRNA. Since low copy number was less reliable, the miRNAs with more than 10 copies were reserved for subsequent analysis. In order to compare miRNA expressions across databases, the total copy number of each sample was normalized to 1,000,000 [16, 17]. According to some reports on multiple isomiRs from a given miRNA locus [18, 19], isomiRs were also comprehensively surveyed. Sequences that matched the pre-miRNAs in the mature miRNA region ±4 nt (no more than 1 mismatch) were defined as isomiRs.

### 2.4. Quantitative Validation of miRNAs by qRT-PCR

We performed quantitative RT-PCR (qRT-PCR) to validate the expression levels of miRNAs analyzed by NGS. The reverse transcription reaction was carried out with AMV reverse transcriptase (Takara, Japan) at 42°C for 60 min and 75°C for 15 min in a total volume of 20 *μ*L. The universal RT primer was 5′-GTCGTATCCAGT GCAGGGTCCGAGGTATTCGCACTGGATACGACNNNNNN-3′. The quantitative PCR amplification was performed with SYBR Premix (Takara, Japan). The forward primers were miRNAs-specific, and the universal reverse primer was GTGCAGGGT CCGAGGT.U6 snRNA was used as internal reference. The reaction was incubated in ABI 7500 PCR system at 95°C for 5 min, followed by 40 cycles of 95°C for 15 s and 55°C for 45 s in a total volume of 10 *μ*L. The cycle threshold (Ct) values were calculated by automatic Ct setting of 7500 system SDS software v1.4 (Applied Biosystems, USA).

### 2.5. Prediction and Functional Analysis of Target mRNA

We used the starbase v2.0 to predict the target gene of miRNAs verified by qRT-PCR [20, 21]. The target genes were obtained by the intersection of three prediction software programs, which were TargetScan, PicTar, and miRanda based on the starbase. Then we used the Gene Ontology (GO) database for function enrichment [22, 23]. In addition, DNA intelligent analysis (DIANA) software was used for pathway identification based on Kyoto Encyclopedia of Genes and Genomes (KEGG) database [24].

### 2.6. Statistical Analysis

We adopted the log_2_ method to transform the normalized reads, and the expression patterns of the sequencing data were visualized by hierarchical clustering. The *p* value of differential expression of miRNAs was calculated based on Poisson's distribution [25] and the threshold of *p* value was determined by false discovery rate (FDR) [26]. The average Ct for each triplicate from qRT-PCR was used for analysis. Fold change in miRNA expression was calculated by ΔΔCt, normalized with ΔCt = AvgCt_miRNA_ − AvgCt_U6_ [27]. A comparison between two groups was performed by using the *t*-test. *p* values of <0.05 were considered to indicate statistically significant differences.

## 3. Results

### 3.1. Results of miRNA Sequencing

Based on Ion Torrent sequencing platform, the total RNAs of the 3 pooled samples from 9 paired AC tissues were characterized. In each pooled sample, more than 50% reads were annotated to miRNAs and ncRNAs database (data not shown). In this study, we detected 259, 401, and 389 known miRNAs in AC tumor, adjacent, and normal tissues, with 65, 95, and 34 novel mature miRNAs detected in the 3 pooled samples, respectively (Table 1). In addition, the sequences mapped with reference genome, on average 89.72% reads, were mapped to the reference human genome (data not shown).

**Figure 1:**
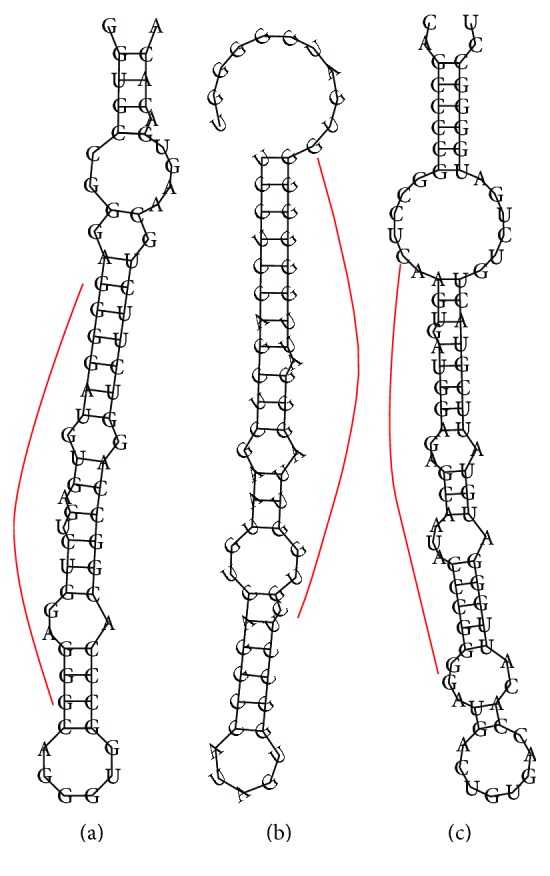
Predicted stem-loop structures of precursors. These 3 precursors had the ability to be processed to form the 3 novel miRNAs in Table 2. The red curve indicated the location that could be processed to miRNAs. (a), (b), and (c) represent tumor, adjacent, and normal control tissues, respectively.

To confirm the existence of miRNA among our sequencing miRNAs, we performed a prediction of the miRNA precursor hairpin structure. The novel miRNAs which had the highest expression levels in each pooled sample were shown in Table 2. The secondary structure of their precursor (Figure 1) had the characteristic of stem-loop hairpin structure, and mature miRNA was far from loops and bulges.

**Figure 2:**
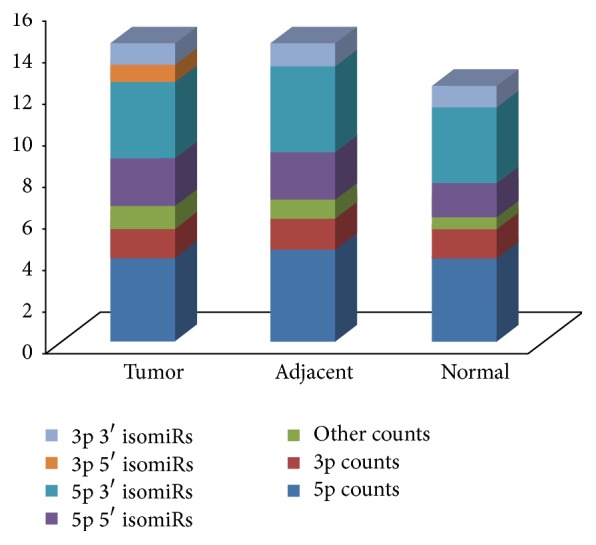
All isoform counts of miR-21 are in 3 pooled samples. More 5p isomiRs and 3′ isomiRs were found in miR-21 compared to 3p isomiRs and 5′ isomiRs, respectively. The *y*-axis meant the value of counts as log_10_.

The biological feature of the reads annotated to miRNAs was analyzed afterwards. The length distributions of all miRNAs in the 3 pooled samples focused on 21, 22, and 23 nt. As expected, reads with a length of 22 nt were the most abundant (data not shown). The peak distributions were consistent with the biological feature of mature miRNAs, indicating that mature miRNAs were well enriched during the preparation of miRNA sequencing library. Simultaneously, all the isomiRs were obtained in each pooled sample. Half of the most dominant isomiR sequences had lengths that were different from those of canonical miRNA sequences. Generally, the most dominant isomiR sequence may be longer or shorter than registered miRNA sequence through altering 5′ or 3′ ends, especially for the 3′ ends (data not shown).  Figure 2 illustrated all the isoform profiles of the differential miR-21 which were validated in subsequent experiment (see Sections 3.3 and 3.4).

### 3.2. The Differentially Expressed miRNAs in Tumor, Adjacent, and Normal Tissues

**Figure 3:**
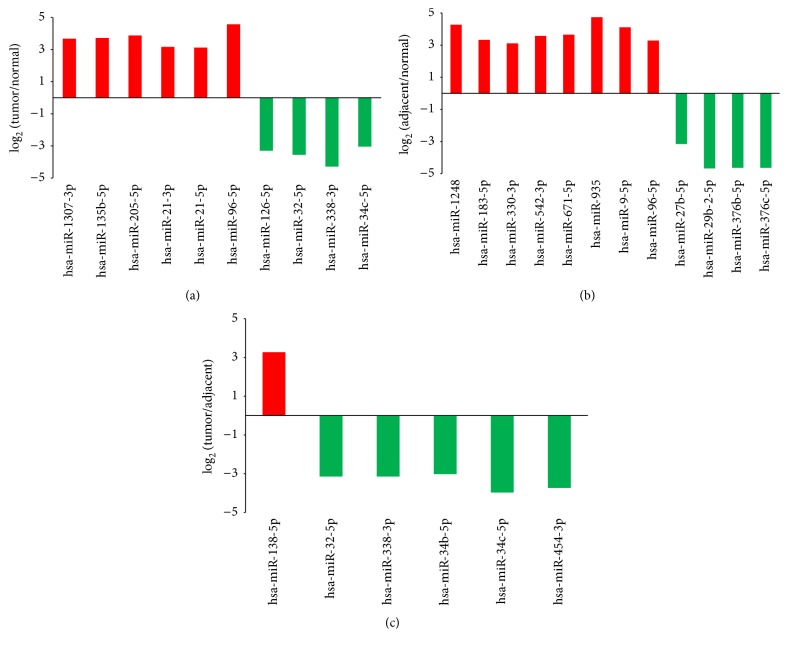
The differential miRNAs with fold change > 3 in tumor, adjacent, and normal tissues. (a) 10 miRNAs differentially expressed between tumor and normal tissues. (b) 12 miRNAs differentially expressed between adjacent and normal tissues. (c) 6 miRNAs differentially expressed between adjacent and normal tissues. The *y*-axis meant the value of fold change as log_2_.

The differential miRNAs were chosen after three procedures. Firstly, we removed the unreliable miRNAs which had low copy number (≤10 reads) in paired samples at the same time. Then the *p* value and FDR value of miRNA reads were calculated, and the miRNAs with FDR < 0.01 were chosen. At last we obtained the applicable miRNAs with 2-fold or more change. So 86, 50, and 76 miRNAs were differentially expressed with 2-fold or greater change in tumor versus normal, adjacent versus normal, tumor versus adjacent tissues, respectively. As shown in Figure 3, with stricter cutoff criteria (fold change > 3), 10 (6 upregulated, 4 downregulated), 12 (8 upregulated, 4 downregulated), and 6 (1 upregulated, 5 downregulated) miRNAs were differentially expressed in tumor versus normal, adjacent versus normal, tumor versus adjacent tissues.

### 3.3. The Gradually Changed miRNAs in Tumor, Adjacent, and Normal Tissues

**Figure 4:**
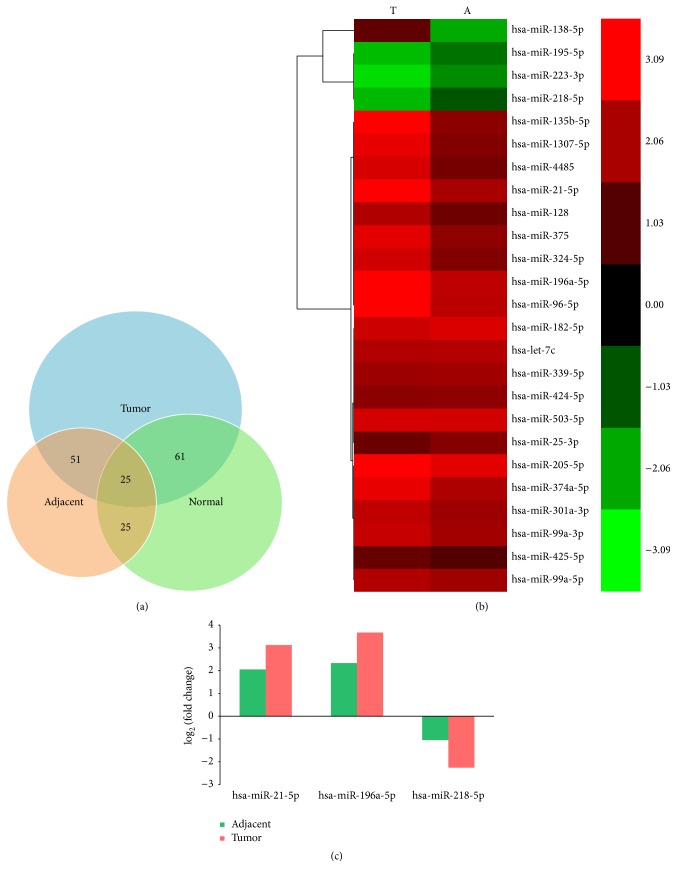
The gradually changed miRNAs with fold change > 2 in tumor, adjacent, and normal tissues. (a) The sets of differential miRNAs among tumor, adjacent, and normal tissues: 25 miRNAs were differentially expressed in both of tumor and adjacent tissues compared to normal control. (b) Hierarchical clustering analysis of 25 miRNAs dysregulated in tumor and adjacent tissues compared to normal control. The column showed each group compared to the normal control. The lines showed the fold change of each miRNA as log_2_, from green to red as from −3 to 3. (c) The 3 miRNAs gradually changed from normal to adjacent to tumor tissues.

In this study, we attempted to find out the miRNAs which gradually changed from normal to adjacent to tumor tissues with 2-fold or greater change and FDR < 0.01 (the normal as control). As shown in Figure 4(a), there were 61 differential miRNAs in tumor but not in adjacent tissues compared to normal control, 25 miRNAs were expressed differentially in adjacent but not in tumor tissues, 51 miRNAs were differentially expressed in both tumor and adjacent tissues but not in control. In addition, 25 miRNAs were differentially expressed in tumor and adjacent tissues at the same time compared to normal control (Figure 4(b)). Among them, miR-21-5p and miR-196a-5p were gradually upregulated from normal to adjacent to tumor tissues and miR-218-5p was gradually downregulated from normal to adjacent to tumor tissues significantly (Figure 4(c)).

### 3.4. qRT-PCR Validation of Differential Expression of Gradually Changed miRNAs

**Figure 5:**
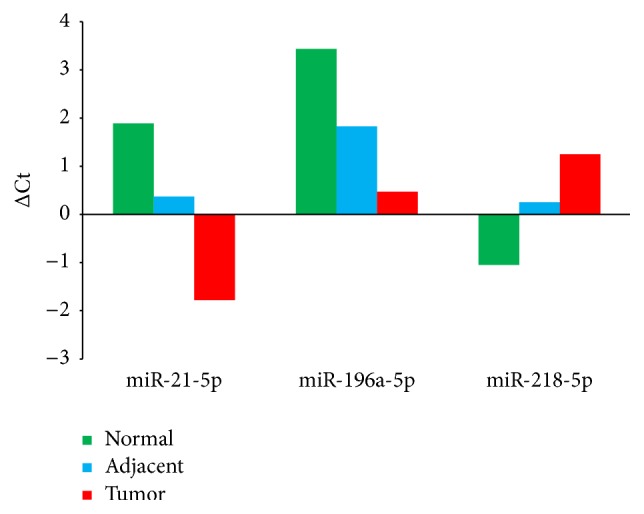
The qRT-PCR results of 3 gradually changed miRNAs. miR-21-5p and miR-196a-5p were gradually upregulated from normal to adjacent to tumor tissues, and miR-218-5p was gradually downregulated from normal to adjacent to tumor tissues significantly (*p* < 0.01, ΔCt = AvgCt_miRNA_ − AvgCt_U6_).

In order to validate the differential expression of these 3 gradually changed miRNAs from NGS results, we performed qRT-PCR to quantify the expression levels of miR-21-5p, miR-196a-5p, and miR-218-5p in 18 paired AC samples (including 9 paired AC samples used in miRNA sequencing). The results suggested that miR-21-5p, miR-196a-5p, and miR-218-5p had consistent gradual changes in normal, adjacent, and tumor samples with sequencing results (*p* < 0.001, Figure 5), and the magnitude of changes differed between qPCR and NGS. The expression levels of these 3 miRNAs quantified by qPCR were shown in Table 3.

### 3.5. Prediction and Functional Analysis of Target mRNA

**Figure 6:**
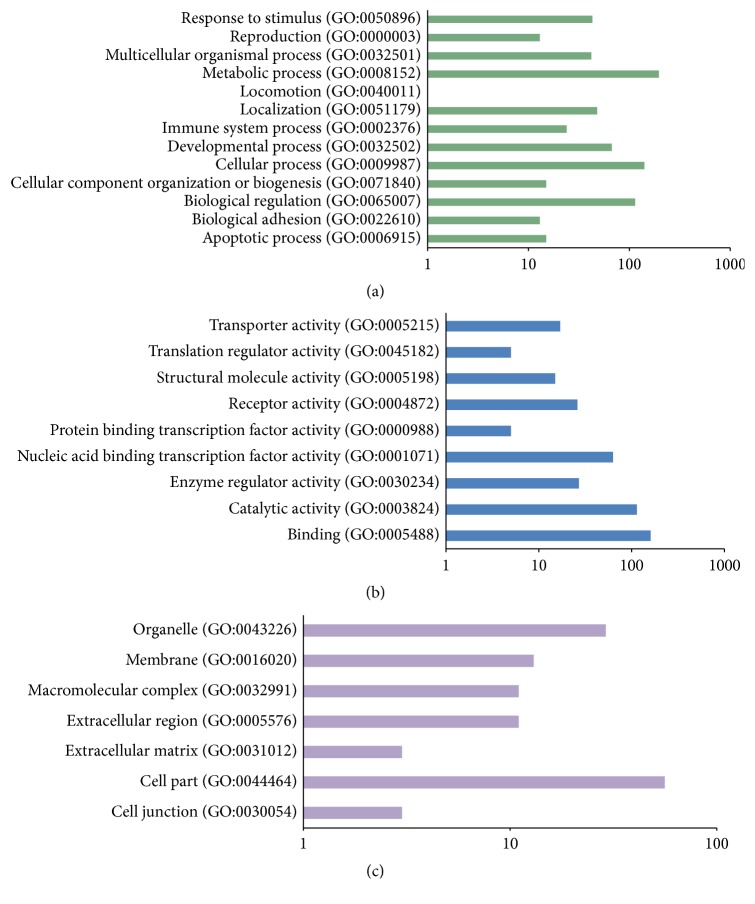
Categories of the Gene Ontology by target genes of 3 miRNAs. (a) Biological process terms were enriched in metabolic process, cellular process, and biological regulation. (b) Molecular function terms were enriched in binding, catalytic activity, and receptor activity. (c) Cell component terms were enriched in cell part, organelle, and membrane.

**Figure 7:**
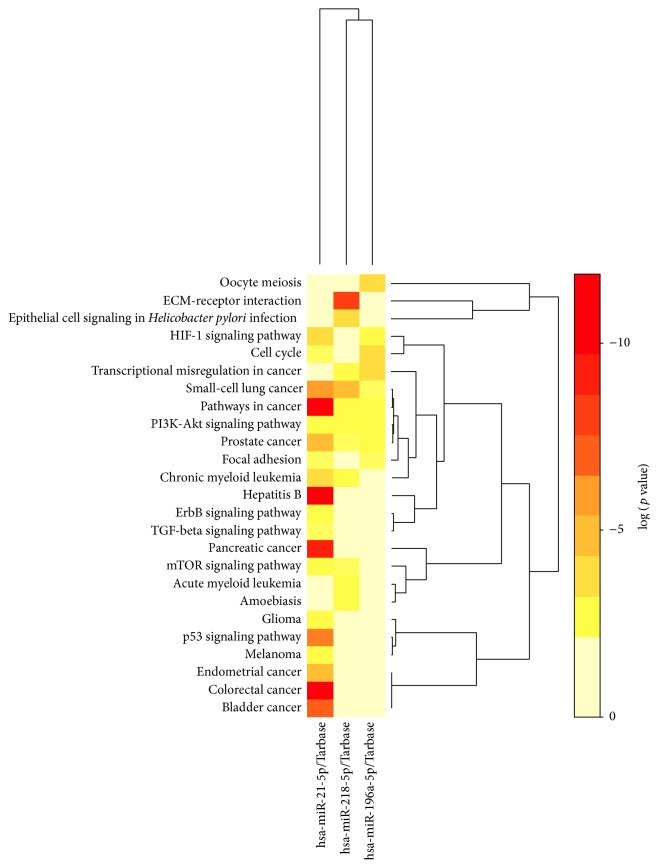
The heatmap of significant KEGG pathways that 3 miRNAs may be involved in. The column showed each pathway and candidate target genes of miRNAs. The color showed red to yellow as log⁡ (*p*  value) from low to high.

The common target genes of these 3 miRNAs were predicted by TargetScan, PicTar, and miRanda based on the starbase. A total of 424 target genes of these 3 differentially expressed miRNAs were predicted and used for GO function enrichment. The significant items of GO biological process, GO molecular function, and GO cell component were shown in Figure 6. We applied the DIANA software to create a rank list of 30 KEGG pathways with the most significant gene enrichment (*p* < 0.05, Table 4). Among them, pathways in cancer ranked first. In addition, plenty of tumor-related pathways were enriched, including non-small-cell lung cancer, colorectal cancer, pancreatic cancer, and prostate cancer. The heatmap of these 3 miRNAs and significant pathways was shown in Figure 7. KEGG pathway showed that miR-21-5p, miR-196a-5p, miR-218-5p, and their potential target genes were involved in the NSCLC pathway. The results indicated that miR-21-5p, miR-196a-5p, and miR-218-5p might serve as tumor oncogene or suppressor gene in NSCLC (data not shown).

## 4. Discussion

Non-small-cell lung cancer (NSCLC) accounts for 80–85% of all kinds of lung cancer, the leading cause of cancer deaths worldwide. Adenocarcinoma is one of the major subtypes of NSCLC. miRNAs have been shown to play roles in the tumorigenesis and the development of lung cancer. However, few studies characterize the profiles of miRNAs in paired tumor, adjacent, and normal tissue samples of adenocarcinoma at the same time.

In this study, we found 25 differential miRNAs coexpressed in tumor, adjacent, and normal tissue samples from AC patients based on next-generation sequencing. Among them, 3 miRNAs displayed gradual changes from normal to adjacent to tumor tissues. Then qRT-PCR was performed to confirm that miR-21-5p and miR-196a-5p were gradually upregulated and miR-218-5p was gradually downregulated from normal to adjacent to tumor tissues significantly. The predicted target genes of these 3 miRNAs may regulate in several tumor-related KEGG pathways, including NSCLC, colorectal cancer, pancreatic cancer, and chronic myeloid leukemia. Therefore, pathways in cancer ranked first in all of the enriched KEGG pathways. In particular, miR-21-5p might target several genes in NSCLC pathway, including EGFR, ERBB2, E2F, CDK4, and CDK6. Moreover, miR-21-5p, miR-196a-5p, and miR-218-5p were also involved in ErbB signaling pathway, PI3K-Akt signaling pathway, and cell cycle.

For the first time we have characterized the miRNA profiles in tumor, adjacent, and normal tissues from adenocarcinoma patients at the same time. The results showed and validated that miR-21-5p and miR-196a-5p were gradually upregulated, and miR-218-5p was gradually downregulated from normal to adjacent to tumor tissues. These indicated that miR-21-5p and miR-196a-5p might serve as oncogene, and miR-218-5p might act as tumor suppressor gene in lung cancer. However, we need to increase the depth of sequencing to validate the results and gain more data of miRNAs in the future study.

miR-21-5p was comprehensively studied in lung cancer by many researchers. Seike et al. found that aberrantly increased expression of miR-21 was enhanced by activated EGFR signaling pathway and played a significant role in lung carcinogenesis in never-smokers [28]. Markou et al. revealed that mature miRNA-21 was significantly upregulated in NSCLC patients and was an independent negative prognostic factor for overall survival in NSCLC [29]. Xu et al. showed that miR-21 regulated cellular proliferation, invasion, migration, and apoptosis by targeting PTEN, RECK, and Bcl-2 in lung squamous carcinoma [30]. However, miR-218-5p and miR-196a-5p were little studied in lung cancer. miR-218 was deleted and downregulated in lung squamous cell carcinoma and may be a strong candidate tumor suppressing miRNA potentially involved in lung cancer [31]. Song et al. found that miR-218 suppressed the growth of lung carcinoma by reducing MEF2D expression [32]. Liu et al. showed that miR-196a promoted NSCLC cell proliferation and invasion through targeting HOXA5 [33]. Although the differential expression levels of the 3 miRNAs were approximate to our results in the previous studies, we validated their gradual changes in paired tumor, adjacent, and normal tissues of lung adenocarcinoma for the first time. The gradual changes may verify the function of miR-21-5p, miR-196a-5p, and miR-218-5p as oncogene or suppressor gene more accurately. As little existing research has studied the profiles and functions of miR-196a-5p and miR-218-5p in lung cancer, we will further validate their regulatory roles in lung cancer in future research.

In conclusion, we characterized the differential expression of miRNAs in paired tumor, adjacent, and normal tissue samples of lung adenocarcinoma based on NGS, and we found and validated the gradual changes of miR-21-5p, miR-196a-5p, and miR-218-5p from normal to adjacent to tumor tissues. These 3 miRNAs may regulate as oncogene or suppressor gene in tumorigenesis and progression of lung adenocarcinoma. Our research provided several miRNA biomarkers for lung adenocarcinoma and may be useful for lung adenocarcinoma diagnosis and the study of pathology in lung cancer.

## Figures and Tables

**Table 1:** The numbers of detected miRNAs in 3 pooled samples.

Sample	Known miRNAs	Novel miRNAs
AC tumor	259	65
AC adjacent	401	95
AC normal	389	34

**Table 2:** The novel miRNAs with the highest expression levels in each pooled sample.

Sample	Novel miRNA	Arm	Sequence of miRNA	Sequence of most abundant taq	Sequence of precursor
AC tumor	xxx-1-5p	5p	GGGGATGTGAGTCTGGAGGG	GGGGATGTGAGTCTCGAGTG	GGTGCCGGGAGGGGATGTGAGTCTGGAGGGCAGGGTGGCCCACGGCCAGGTCTTCTGCAAGTGACACA

AC adjacent	xxx-2-3p	3p	GTGGTTAGGGATTGGGGGGG	GTGGTTAGGGATTCGGCGCT	TCCTCCAGCTCGAATGTCAGGCCATAGTGGCCTCGTGGTTAGGGATTGGGGGGGTGATGGGGGT

AC normal	xxx-3-5p	5p	CAAGTGATGGAGAGCAATACCCGG	TCAAGTGATGGAGAGCAAT	CAGCCCCGGCCTCAAGTGATGGAGAGCAATACCCGGGGATGACTGTGACCACATTGGGATGTATTCGTACTGTCTGATGGGGCCT

**Table 3:** The expression levels of 3 miRNAs in 18 pairs of tumor, adjacent, and normal tissue samples of adenocarcinoma quantified by qRT-PCR.

miRNAs	Mean ± SD in tumor samples	Mean ± SD in adjacent samples	Mean ± SD in normal samples	*p* value (tumor versus normal, adjacent versus normal, and tumor versus adjacent)
miR-21-5p	−1.78 ± 0.12	0.37 ± 0.08	1.89 ± 0.11	<0.001
miR-196a-5p	0.47 ± 0.09	1.83 ± 0.06	3.44 ± 0.04	<0.001
miR-218-5p	1.25 ± 0.14	0.35 ± 0.05	−1.05 ± 0.09	<0.001

**Table 4:** KEGG pathway of predicted target genes.

KEGG pathway	Number of target genes	*p* value
Pathways in cancer	35	6.33*e* − 17
Small-cell lung cancer	15	1.04*e* − 11
Hepatitis B	19	6.85*e* − 11
Colorectal cancer	11	9.08*e* − 10
Pancreatic cancer	13	1.57*e* − 09
Prostate cancer	12	1.50*e* − 07
p53 signaling pathway	11	1.71*e* − 07
Bladder cancer	8	7.08*e* − 07
Chronic myeloid leukemia	11	8.30*e* − 07
PI3K-Akt signaling pathway	25	2.21*e* − 06
HIF-1 signaling pathway	13	2.45*e* − 06
Focal adhesion	17	6.38*e* − 05
mTOR signaling pathway	8	0.00020
Transcriptional misregulation in cancer	14	0.00034
Endometrial cancer	7	0.00040
Cell cycle	13	0.00069
Melanoma	8	0.00113
Valine, leucine, and isoleucine biosynthesis	1	0.00337
Glioma	7	0.00337
ErbB signaling pathway	6	0.00337
TGF-beta signaling pathway	9	0.00347
Acute myeloid leukemia	6	0.00406
Amoebiasis	9	0.00846
Synthesis and degradation of ketone bodies	2	0.01976
Wnt signaling pathway	11	0.01987
Non-small-cell lung cancer	6	0.02290
ECM-receptor interaction	7	0.02443
Chagas disease (American trypanosomiasis)	8	0.02446
*Herpes simplex* infection	13	0.02446
Hypertrophic cardiomyopathy (HCM)	7	0.02446
